# Identification and characterization of tissue resident memory T cells in malignant pleural effusions associated with non-small cell lung cancer

**DOI:** 10.1093/immhor/vlaf013

**Published:** 2025-04-24

**Authors:** Caitlin M Tilsed, Joshua Brotman, Shaun O’Brien, Brennan Lee, Edmund Moon, Steven M Albelda

**Affiliations:** Pulmonary, Critical Care, and Allergy Division, Department of Medicine, Perelman School of Medicine at the University of Pennsylvania, Philadelphia, PA, United States; Pulmonary, Critical Care, and Allergy Division, Department of Medicine, Perelman School of Medicine at the University of Pennsylvania, Philadelphia, PA, United States; Informatics and Predictive Sciences, Mechanisms of Cancer Resistance, Bristol Myers Squibb, Cambridge, MA, United States; Informatics and Predictive Sciences, Mechanisms of Cancer Resistance, Bristol Myers Squibb, Cambridge, MA, United States; Pulmonary, Critical Care, and Allergy Division, Department of Medicine, Perelman School of Medicine at the University of Pennsylvania, Philadelphia, PA, United States; Pulmonary, Critical Care, and Allergy Division, Department of Medicine, Perelman School of Medicine at the University of Pennsylvania, Philadelphia, PA, United States

**Keywords:** lung cancer, pleural effusion, T cells, tissue resident memory T cells

## Abstract

Tissue resident memory T cells (T_RM_) play a critical role in cancer immunity and their presence in solid tumors is associated with improved prognosis and response to therapy. Although T_RM_ have been identified and their function characterized in lung cancers, little is known regarding T_RM_ outside of a tissue context, such as within malignant pleural effusions (MPE). As MPE are routinely drained and collected to manage symptoms, analysis of this fluid can provide an insight into the peri-tumoral environment. In this study, we performed flow cytometry and single cell RNAseq (scRNAseq) on MPE associated with non-small lung cancer and examined the phenotype and function of T_RM_. We found that 14% of CD8^+^ T cells and 6% of CD4^+^ T cells were T_RM_, as defined by the phenotype of CD45RO^+^CCR7^-^CD62L^-^ and expressing 1 or both of CD69 and CD103. The scRNAseq revealed distinct clusters expressing T_RM_-associated genes including ITGAE and CD49A and lacking expression of SELL, CCR7, and IL7RA. T_RM_ did not differ from other memory T cell subsets, such as T central memory (T_CM_) and T effector memory (T_EM_) cells, in expression of the inhibitory markers PD-1, TIGIT, and CD39. When TRM function was assessed by measuring the production of IFN-γ, TNF-α, and CD107a after stimulation with αnti-CD3 antibodies in vitro, T_RM_ had comparable function to T effector cells (T_E_), indicating that despite expression of exhaustion markers these cells retained effector function. Finally, we found that CD69 expression, and not CD103 expression, on T_RM_ was associated with production of effector cytokines.

## Introduction

Over the past decade, it has become increasingly apparent that in addition to circulating lymphocytes, a tissue-resident subset of T cells (T_RM_) play an important role in orchestrating local secondary defense responses.^1^^,^^2^ T_RM_ are antigen-experienced memory T cells that can be immediately reactivated in peripheral tissues and can thus provide a rapid acquired immune response to infections. In addition, an important role of T_RM_ in cancer immunity has become established over recent years.^3^ The presence of T_RM_ in many solid cancers has been associated with an improved prognosis ^4–6^ and response to therapy.^7^^,^^8^

The identification of T_RM_ relies on the presence of a number of markers in different combinations.^2^^,^^9^ Similar to T effector memory cells (T_EM_), T_RM_ are CD45RO^+^, CD44^hi^, L-selectin (CD62L)^lo^, and CCR7^lo^. In contrast to T_EM_, markers that are reported to be more uniquely upregulated in T_RM_ include the integrin complex called CD103 (which binds to E-cadherin), the integrin CD49a (which binds to collagen), CD69 (which inhibits the S1PR1-mediated egress of T cells from tissues), the chemokine receptor CXCR6, and regulators of G-Protein signaling 1 and 2 (RGS1, RGS2).^10^^,^^11^ T_RM_ also have increased expression of activation/checkpoint markers such as PD1, CTLA4, Tim3, and TIGIT.^12^ In addition to CD62L and CCR7, T cell markers that are downregulated in T_RM_ include sphingosine 1-phosphate receptor 1 (S1PR1), sphingosine 1-phosphate receptor 5 (S1PR5), and Krueppel-like factor 2 (KFL2).^9^ However, none of the described proteins is sufficient alone to define T_RM_ cells and not all of the markers are present on T_RM_ isolated from various locations. Importantly, expression of the 2 most commonly observed markers, CD69 and CD103 can vary among tissues. For example, CD4^+^ T_RM_ cells in the human dermis lack CD103 expression, whereas those in the epidermis are CD103^+^.^9^^,^^13^ So far, analyses of T_RM_ cells in human tumors have mostly relied on the expression of one (e.g., CD103) or a few markers, but it is likely that different combinations of markers will be present on T cells occupying different anatomic niches and tumor types.

Whereas we^14^ and others^15^ have described the presence and function of T_RM_ in lung cancers, there is very little information about this T cell subset in the large group of cancer patients who have malignant pleural effusions (MPE). MPE are common in patients with thoracic and breast cancers and they have a distinct microenvironment containing immune cells, cytokines, and tumor cells. Since this pleural fluid is routinely drained and collected to manage symptoms, it provides a useful opportunity to gain insight into the peri-tumoral environment since it is adjacent to the primary or metastatic tumor and can be studied as a window into the human solid tumor microenvironment that is often difficult to accesss.^16^ The immune composition of MPE can be a prognostic factor for survival^17–19^ and a predictor for response to immunotherapy,^20^ indicating the importance of characterizing the phenotype and functionality of intrapleural immune cells.

The focus of this investigation is on T_RM_ in MPE associated with non-small cell lung cancer. Although there have been a multitude of studies characterizing the MPE microenvironment using flow cytometry and single cell RNA sequencing (scRNAseq),^16^^,^^21^^,^^22^ there has been little investigation into the presence and function of T_RM_ within this environment. We were able to identify only 1 study that specifically identified T_RM_ within malignant effusions (pleural and peritoneal) using flow cytometry.^22^ In the CD8 compartment, the authors noted that a majority of cells expressed either CD69, CD103, or both markers. The response of the T_RM_ cells to strong stimulation was less than that observed in blood T cells, but they did not compare the response of T_RM_ with other types of effusion T cells. Therefore, additional studies are needed to characterize the presence of T_RM_ within MPE, and to study their functionality, phenotype, and contribution to the anti-tumor immune response.

In this study, we phenotyped the CD8^+^ and CD4^+^ T cell subsets found in lung cancer MPE using flow cytometry and single cell RNAseq with a focus on T_RM_. We next characterized the effector functionality of each population and then further investigated the differences in T_RM_ that expressed CD103, CD69, or both markers.

## Materials and methods

### Sample collection

Patients undergoing thoracentesis for malignant pleural effusions were consented (Approved Protocol no. 823659 from the University of Pennsylvania Office of Regulatory Affairs) to allow the use of excess fluid not used for diagnostic purposes to be used for research analysis. After collection, MPE samples were centrifuged and red blood cell lysis performed if necessary. Cells were used fresh in experiments.

For the flow cytometry studies, 11 patients were studied (1 patient contributed 2 effusions). All patients had Stage IV adenocarcinoma of the lung. The average age was 68 yrs (range: 52 to 84 yrs), 33% were male, 67% were white, and 66% were current or former smokers. The therapies at the time of the thoracentesis for analyses are shown in Table S1. Three patients had received no treatment, 1 had received a PD1 antibody in combination with carboplatin and pemetrexed, 1 had received a PD1 antibody with a Cox2 inhibitor, 1 received abraxane, and 4 had received Osimertinib. One patient had their effusion analyzed before treatment and then after receiving PD1 antibody in combination with carboplatin and pemetrexed.

For the scRNAseq studies, 3 patients were studied. All were females, ages 62, 63, and 72, and all had metastatic Stage IV adenocarcinoma. Two patients had been treated with Osimertinib and one with pembrolizumab.

### Flow cytometry

For MPE characterization, samples were stained fresh after collection. Samples were first stained with live/dead UV for 15 min in the dark at room temperature (RT) for dead cell exclusion followed by a wash in PBS + 2%FCS. Antibodies for cell surface staining were suspended in PBS + 2%FCS and incubated for 30 min at RT. Cells were washed once again and data acquired using the BD LSRFortessa and analyzed using FlowJo (BD Biosciences), using the facilities of the Penn Cytomics & Cell Sorting Shared Resource Laboratory.

For restimulation assays, fresh MPE were resuspended in R10 media (RPMI, 10% PBS, 1% pen/strep, 1% L-glutamine). Samples were incubated with GolgiStop (BD Biosciences, 0.66 μl/ml) and GolgiPlug (BD Biosciences, 1 μl/ml) and stimulated with 0.5 μg/ml plate-bound anti-human CD3 for 18 h (OKT3, Biolegend). After live/dead and surface antibody staining as described above, cells were fixed and permeabilized for 10 min at RT using the Foxp3/Transcription Factor Staining Buffer Set (eBioscience). Intracellular antibodies were suspended in Permeabilization Buffer (eBiosciences) and incubated for 30 min at RT. Samples were washed in permeabilization buffer and analyzed.

### Single cell RNAseq

MPE samples were shipped to Bristol Myers Squibb. Single cell Libraries were constructed using the Chromium Next GEM Single Cell 3′ Reagent kits from 10X, and single cell RNA sequencing was performed on an Illumina NextSeq 550. Data were analyzed in R using Seurat (Version 5).^23^ The number of genes detected per cell, number of unique molecular identifiers and the percentage of mitochondrial genes were plotted, empty droplets excluded, and outliers removed to filter out dead cells and doublets. Counts from each individual patient were then integrated and downstream PCA, cluster identification and UMAP visualization performed using Seurat. Clusters were labeled using SingleR^24^ to generate broad phenotypes and then manually labeled as T_E_, T_N_, T_RM_, T_EM_, and T_CM_ based on the expression of key genes based on previous studies.^25^

### Statistics

Statistical details for each experiment can be found in the respective figure legends. Statistical analysis for flow cytometry experiments were performed in Graphpad Prism V8 and significance was defined as *P* < 0.05. Analysis of single cell RNAseq data was analyzed as described in respective methods.

## Results

### Both CD8^+^ and CD4^+^ T_RM_ can be identified in MPE

**Figure 1.**
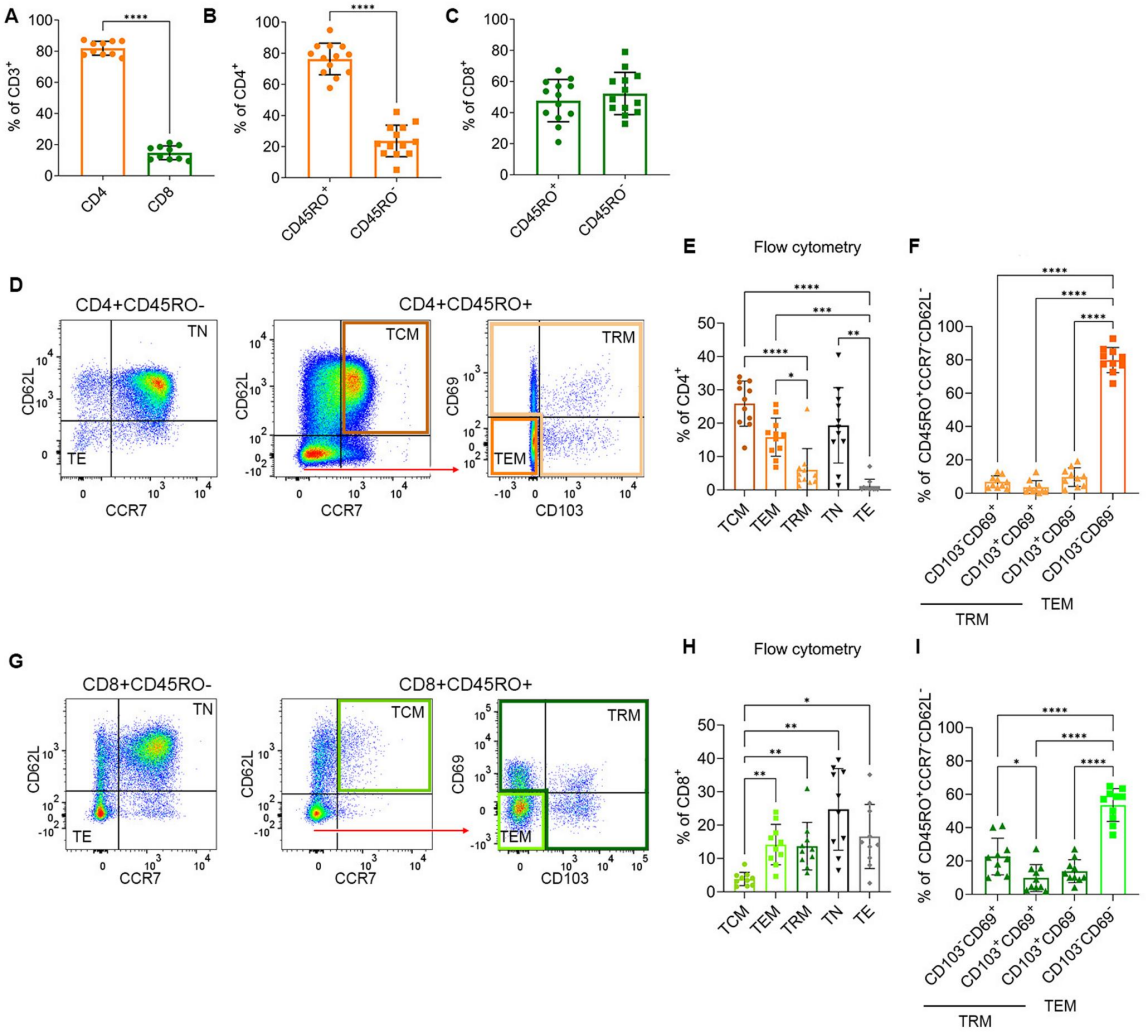
CD8^+^ and CD4^+^ tissue resident memory T cells are found in malignant pleural effusions. (A) Percentage of CD4^+^ and CD8^+^ T cells of CD3^+^ in malignant pleural effusions (MPE). (B and C) CD45RO^+^ and CD45RO^-^ of CD4^+^ (B) and CD8^+^ (C). (D) Representative flow plots for the gating of CD4^+^ T cell subsets. CD4^+^ cells were separated into CD45RO^−^ and CD45RO^+^. T_N_ were classified as CD45RO^-^CCR7^+^CD62L^+^, T_E_ as CD45RO^−^CCR7^−^CD62L^−^ and T_CM_ as CD45RO^+^CCR7^+^CCR7^+^. Within the CD45RO^+^CCR7^−^CD62L^−^ population, cells were gated on CD103 and CD69. T_EM_ were identified as CD45RO^+^CCR7^−^CD62L^−^CD103^−^CD69^−^ and T_RM_ broadly as CD45RO^+^CCR7^−^CD62L^−^ and expressing one or both of CD103 or CD69. (E) Proportion of CD4^+^ subsets as characterized using flow cytometry. (F) Proportion of CD103 and CD69 expressing T cells within the CD45RO^+^CCR7^−^CD62L^−^ population. (G) Representative flow plots for the gating of CD8^+^ T cell subsets, gated as described above. (H) Proportion of CD8 subsets as characterized using flow cytometry. (I) Proportion of CD103 and CD69 expressing T cells within the CD45RO^+^CCR7^−^CD62L^−^ population. Significance was determined using a paired T test or a 1-way ANOVA adjusted for multiple comparisons, *P* < 0.05*, *P* < 0.001**, *P* < 0.0001***, *P* < 0.00001****.

Little is known about the frequency and phenotype of T_RM_ in MPE. Therefore, we first characterized the T cell subsets found in 12 MPE from patients with lung cancer using flow cytometry (Fig. S1). Consistent with previous studies, MPE consisted predominantly of T cells with the ratio of CD4 to CD8 T cells skewed heavily towards CD4^+^ T cells, which represented 80% of the CD3^+^ cells (*P* < 0.0001, Fig. 1A). Within the CD4^+^ population, most cells were CD45RO^+^ memory T cells (*P* < 0.0001, Fig. 1B); in contrast, within the CD8^+^ T cell population, there were similar proportions of CD45RO^+^ (memory) and CD45RO^-^ (naïve) cells (*P* = 0.5571, Fig. 1C).

We next looked to further phenotype the T cells into naïve T cells (T_N_), effector T cells (T_E_), and central memory T cells (T_CM_)^26^ as well as tissue resident T cells (T_RM_) based on expression of CD45RO, CCR7, CD62L, CD69, and CD103 (Fig. S1). T_N_ were classified as CD45RO^-^ CCR7^+^CD62L^+^, T_E_ as CD45RO^-^CCR7^-^CD62L^−^, T_CM_ as CD45RO^+^CCR7^+^CD62L^+^, T_EM_ as CD45RO^+^CCR7^−^ CD62L^−^CD69^−^CD103^−^ and T_RM_ as CD45RO^+^CCR7^−^CD62L^−^ and expressing one or both of CD69 and CD103.

CD4^+^ T cells showed relatively few T_E_ and T_RM_ (<6%), with T_CM_ (25%), T_EM_ (16%), and T_N_ (19%) being the predominant T cell subtypes found in MPE (Fig. 1D and E). Of the CD45RO^+^CD62L^-^CCR7^−^ cells, the expression of CD103 and CD69 was low, indicating few T cells had a T_RM_-like phenotype and the majority were T_EM_ (Fig. 1F).

The phenotype of the CD8 T cells was quite different. T_N_ formed the highest proportion of CD8^+^ T cells in MPE (24%), T_CM_ the smallest (4%), and T_EM_ (14%), T_RM_ (14%), and T_E_ (17%) were all found in similar abundance (Fig. 1G and H). About half of the CD45RO^+^CCR7^−^CD62L^−^ expressed either CD103 or CD69 (Fig. 1I). Within the populations classified as T_RM_ (expressing one or both of CD103 and CD69) the majority expressed only CD69, while those expressing both CD69 and CD103, and the CD103 single positives were found in similar frequencies (Fig. 1I).

### Memory CD8^+^ T cells express high levels of PD1 and TIGIT in MPE

**Figure 2.**
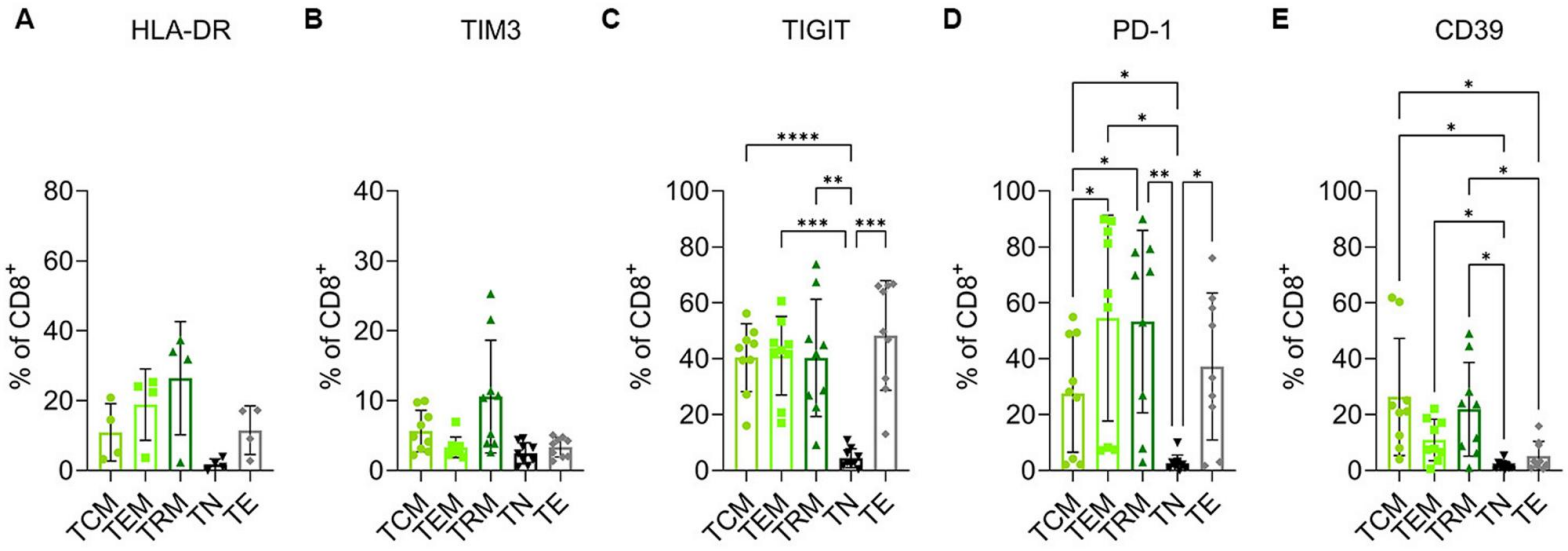
T cells in MPE express high levels of TIGIT and PD-1. (A–E) Expression of HLA-DR (A), TIM3 (B), TIGIT (C), PD-1 (D) and CD39 (E) on CD8^+^ T cell subsets as determined by flow cytometry. *n* = 4 for HLA-DR, *n* = 9 for all other markers. Significance was determined using a one-way ANOVA adjusted for multiple comparisons, *P* < 0.05*, *P* < 0.001**, *P* < 0.0001***, *P* < 0.00001****.

Focusing on these CD8 T cell subsets, we next compared the expression of activation and inhibitory receptors. The expression of the activation marker HLA-DR was negligible on the T_N_ and ranged between 10% and 25% among the other groups (Fig. 2A). These differences did not reach significance, but T_RM_ had the highest level of expression (26%). Expression of Tim3 (3% to 10%, Fig. 2B) were generally low with no significant differences among T cell subtypes. Approximately 50% of T_EM_, T_RM_, T_CM_, and T_E_ expressed TIGIT, all at a significantly higher proportion than T_N_, which had minimal TIGIT positivity (Fig. 2C).

All subsets expressed PD-1 at a higher frequency (25-55%) compared to T_N_ (3%) (Fig. 2D). A significantly larger proportion of T_EM_ (54%; *P* = 0.01) and T_RM_ (53%; *P* = 0.01) expressed PD-1 compared to T_CM_ (27%) (Fig. 2D). There were no significant differences in PD-1 expression between T_EM_, T_RM_ and T_E_.

A subset of T_CM_ (26%), T_EM_ (11%) and T_RM_ (22%) expressed CD39 (a marker of antigen exposure and exhaustion^27^) at a higher frequency then T_N_ (*P* = 0.045, *P* = 0.024 and *P* = 0.033 respectively) and a higher proportion of T_CM_ (*P* = 0.03) and T_RM_ (*P* = 0.043) expressed CD39 then T_E_ (Fig. 2).

These flow cytometry data show that there is a broad range of T cell phenotypes found in MPE, but that a sizable percentage (14%) of CD8 T cells are T_RM_. The proportion of CD4^+^ T_RM_ is quite small. In general, the phenotype of CD8^+^ T_RM_ cells resembled that of T_CM_ and T_EM_.

### T_RM_ can be detected in MPE using single cell RNA sequencing and express most canonical T_RM_ associated genes

**Figure 3.**
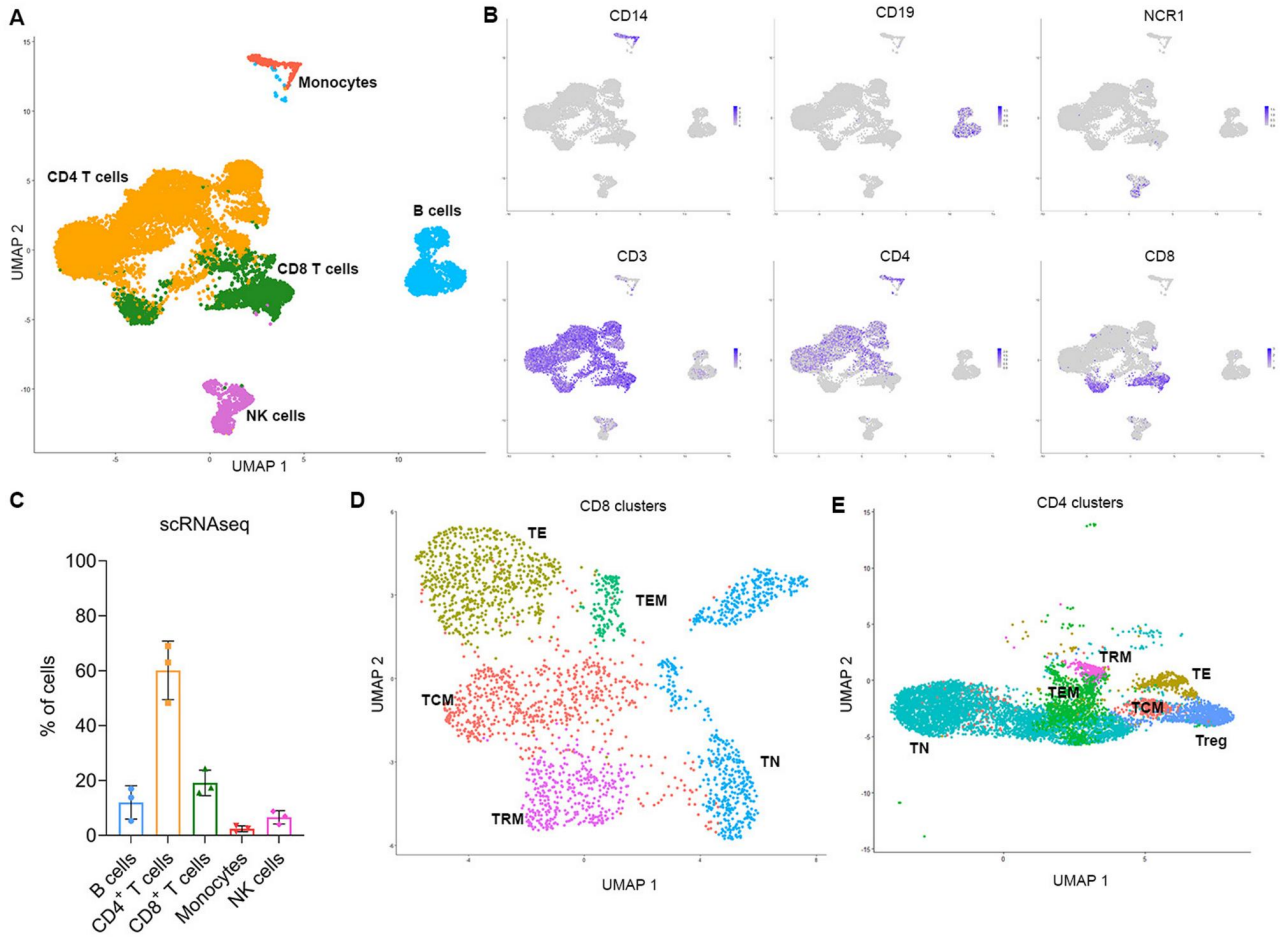
Clusters with gene expression profiles that match a T_RM_ phenotype can be identified within MPE. (A) UMAP clustering of cells from the MPE of 3 patients with lung cancer. (B) Individual plots show the expression of key phenotyping makers used to label cell clusters. (C) Proportions of immune cell populations in the MPE samples. (D) CD8 T cell clusters identified within the MPE samples. (E) CD4 T cell clusters identified within the MPE samples.

We next used single cell RNAseq to further characterize T cells within the MPE with a focus on cells that had a T_RM_-like gene signature.^25^^,^^28^ 3900 cells per patient, for a total of 11,700 cells across 3 lung cancer MPEs were analyzed. Using UMAP clustering, the cells were clustered and then labeled based on their expression of key genes using SingleR^24^ (Fig. 3A and B). Consistent with the flow cytometry data, MPE primarily consisted of CD3^+^ T cells, skewing towards a higher ratio of CD4^+^ T cells to CD8^+^ T cells (Fig. 2C). Monocytes, B cells and NK cells were present in MPE, albeit in small frequencies (Fig. 2C).

**Figure 4.**
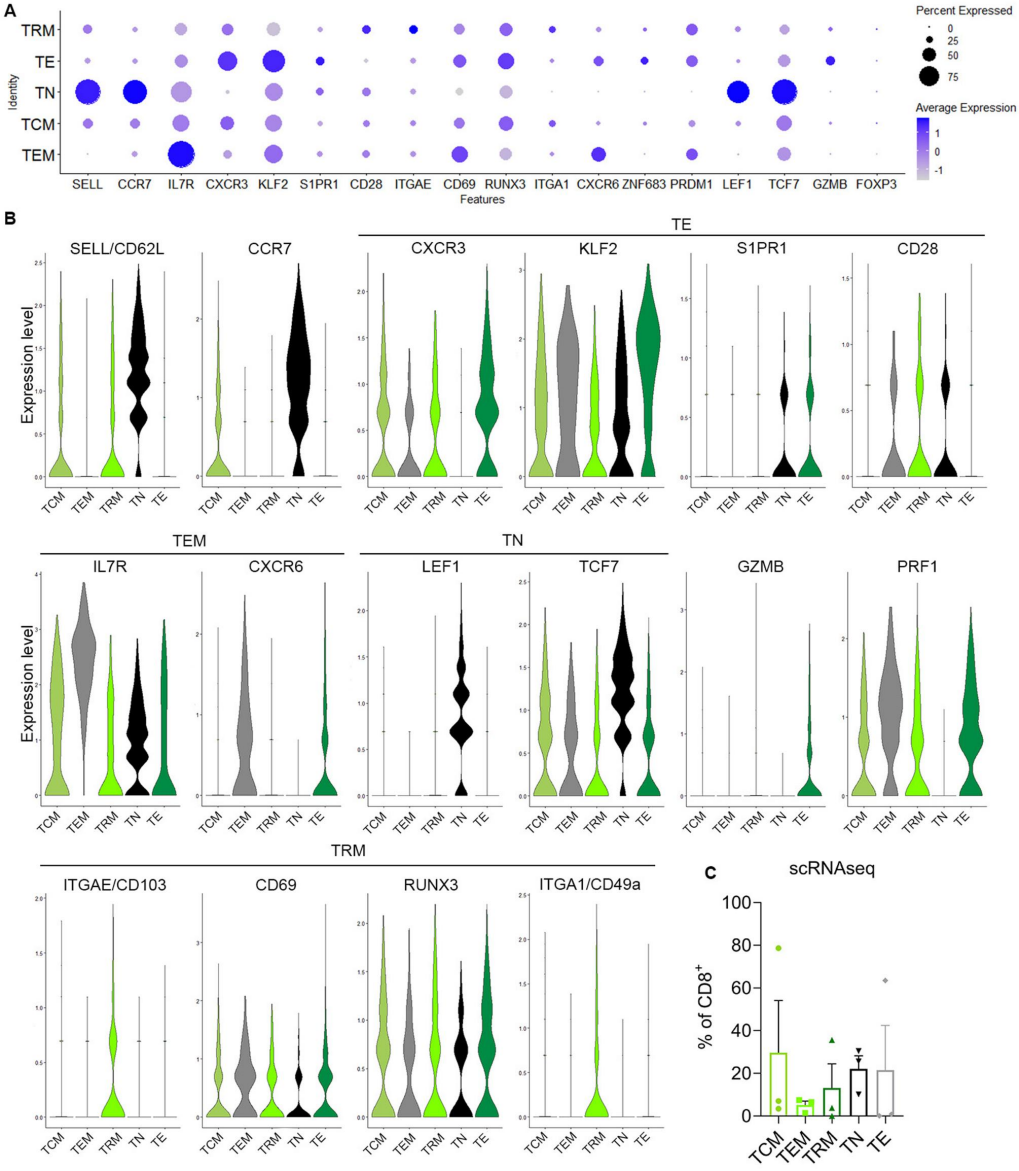
CD8^+^ T_RM_ in MPE has a distinct gene expression profile using scRNAseq. (A) Dot plot showing the expression of key T_RM_, T_E_, T_N_, T_CM_, and T_EM_ genes within the CD8^+^ T cell clusters and the frequency of cells expressing these genes. (B) Violin plots showing expression of a selection of genes within the CD8^+^ T cell clusters. (C) Proportion of CD8^+^ T cells that are T_CM_, T_EM_, T_RM_, T_N_, and T_E_ using scRNAseq. *n* = 3.

We next focused on the CD8^+^ T cells and performed unsupervised clustering, which identified 5 distinct CD8 populations (Figs 3D and 4A and B). Two clusters had high levels of CD62L and CCR7. The cluster designated as T_N_ also had high expression of LEF1 and TCF7. The cluster designated as T_CM_ also expressed intermediate IL7R and TCF and lacked expression of S1PR1 and CD28.^28^ The remaining 3 clusters had low levels of CD62L and CCR7. The cluster designated as T_EM_ expressed high levels of IL7R and low expression of T_CM_ genes. The cluster designated as T_E_ expressed granzyme-B, CXCR3, KLF2 and S1PR1, with low CD28. The remaining cluster expressed high levels of ITGAE/CD103, ITGA1/CD49a with low levels of S1PR, SELL/CD62L, CCR7 and IL7R, indicating a T_RM_-like phenotype.^25^^,^^28^ Interestingly, there were no distinct differences in the expression of CD69, RUNX3, nor PRDM1/BLIMP between our T_RM_ like CD8^+^ T cells and the other T cell subsets, differing from what has been previously reported. Additionally, we detected no expression of CXCR6 or ZNF683/HOBIT on our T_RM_ cluster (Fig. 4A and B) There was some variability in the frequency of each CD8 T cell subset between patients (Fig. 4B).

**Figure 5.**
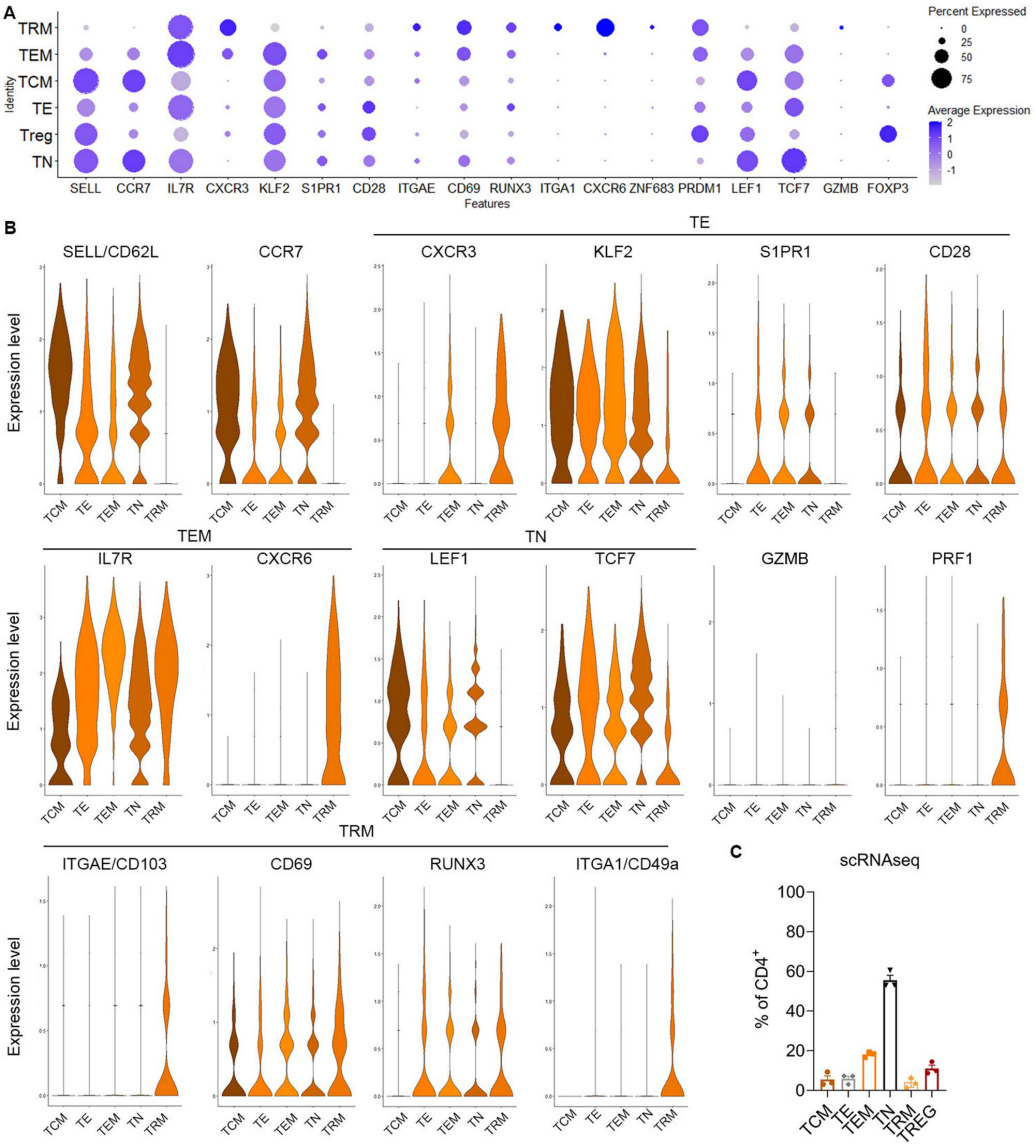
CD4^+^ T_RM_ in MPE express canonical TRM genes as detected using scRNAseq. (A) Dot plot showing the expression of key T_RM_, T_E_, T_N_, T_CM_, and T_EM_ genes within the CD4^+^ T cell clusters and the frequency of cells expressing these genes. (B) Violin plots showing expression of a selection of genes within the CD4^+^ T cell clusters. The Treg cluster was removed for this analysis. (C) Proportion of CD4^+^ T cells that are T_CM_, T_EM_, T_RM_, T_N_, and T_E_ using scRNAseq. *n* = 3.

We also examined the expression of T_RM_ genes within the CD4^+^ T cell clusters (Fig. 3E). The TRM cluster showed a distinct pattern of expression of several genes of interest (Fig. 5A). The CD4^+^ T_RM_ cluster was identified by the lack of SELL/CD62L and CCR7 and the expression of CD103 and CD49a, the latter of which were uniquely expressed on T_RM_ (Fig. 5A and B). Similar to what we observed in the CD8+ T cells, there was no difference in RUNX3 between TRM and the other clusters. However, we did see an increase in CD69, CXCR6, PRDM1/BLIMP and ZNF683 expression by the T_RM_ compared to other CD4^+^ T subsets (Fig. 5A and B), indicating that the CD4_+_ T_RM_ display a more typical gene expression profile compared to their CD8^+^ counterparts. In regard to the other T cell subtypes, 2 clusters expressed high levels of SELL/CD62L and CCR7, which were identified as the T_N_ and T_CM_ clusters and differentiated based on expression of LEF1, TCF7, and S1PR1. The T_EM_ cluster lacked expression of SELL/CD62L and CCR7 and had high expression of IL7R. Interestingly, while the CD8^+^ T_EM_ expressed high levels of CXCR6, this was not seen in CD4^+^ T_EM_. Lastly, the T_E_ cluster did not express SELL/CD62L, CCR7 nor any genes strongly associated with other subtypes. T_N_ were the prominent CD4^+^ cluster as determined using single cell RNA sequencing, making up 60% of the CD4+ T cells, with the other populations ranging from 5% to 20% of the CD4^+^ MPE component (Fig. 5C).

In summary, we were able to detect a T_RM_ like population of CD8^+^ and CD4^+^ T cells in MPE using single cell sequencing based on the high expression of CD103 and CD49a and low expression of S1PR1, CD62L and CCR7. However, in the CD8^+^ T_RM_ we could not detect the expression of some “classical” T_RM_ genes such as CXCR6 or HOBIT and expression of RUNX3 was no different to other T cell subsets. Although CD69 was a useful marker to distinguish T_RM_ from T_EM_ in MPE using flow cytometry, there was no difference in the transcriptional level of CD69 between T cell subsets in the CD8^+^ T cells. CD69 and CXCR6 were able to be detected within the CD4^+^ T_RM_ population.

### MPE T_RM_ have comparable functionality to T_E_, producing CD107a, TNF-α and IFN-γ after restimulation

Little is known about the functional characteristics of MPE T_RM_. One previous study showed that T_RM_ in the MPE were less functional then T_RM_ in the blood;^29^ however, there has not been a direct comparison in functionality between T cell subsets within MPE. To assess this, we first performed restimulation assays using plate-bound anti-CD3 antibodies. We employed surface flow cytometry to assess for the translocation of the degranulation marker CD107a and intracellular flow cytometry to determine the expression of the effector cytokines IFN-γ and TNF-α produced by specific T cell subtypes. Representative flow plots for the restimulation assays are found in the Supplementary Data (Figs S2 and S3).

**Figure 6.**
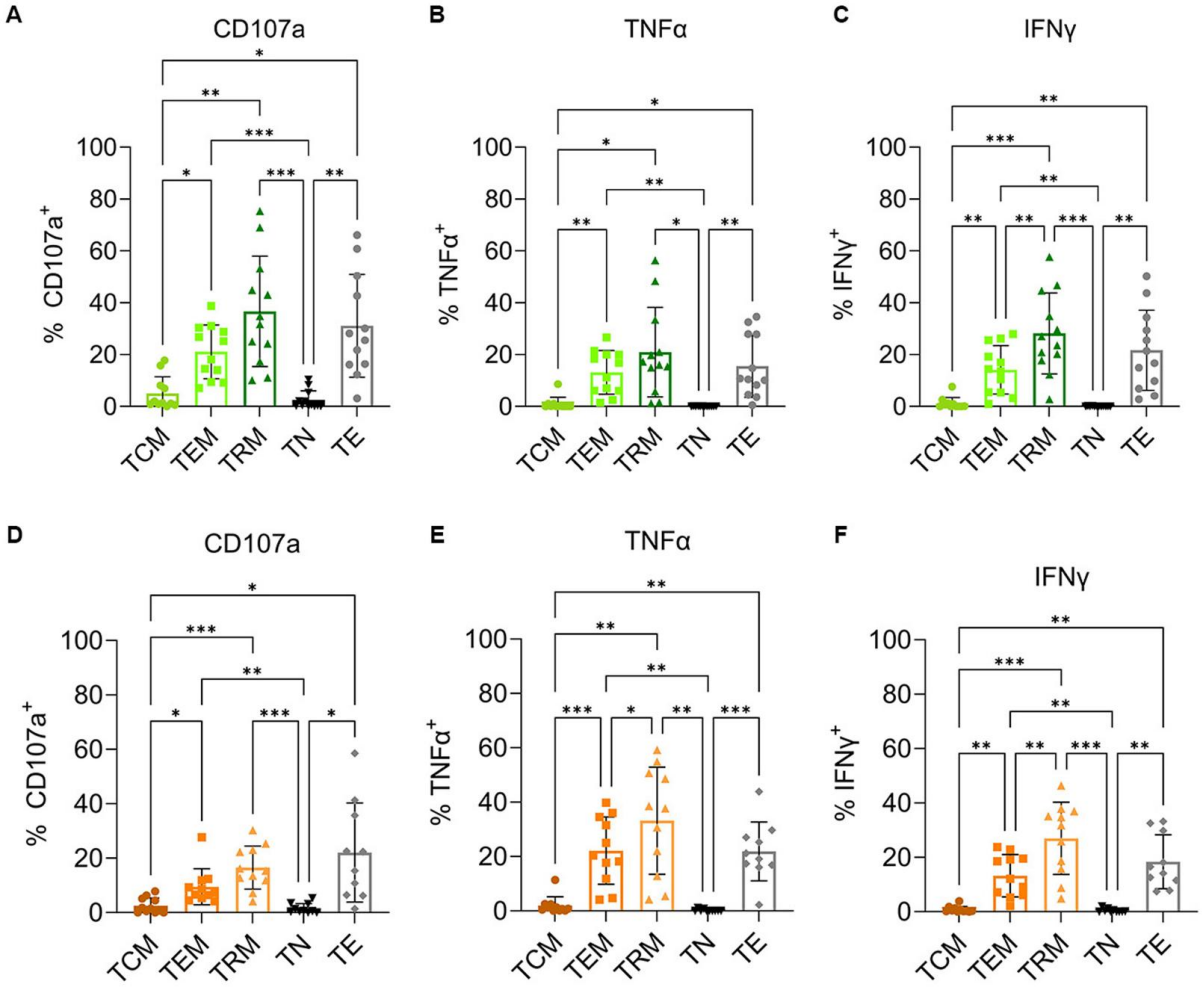
CD8^+^ and CD4^+^ T_RM_ from MPE produce effector cytokines upon ex vivo restimulation at a similar level as T_E_ and T_EM_. (A–C) Proportion of each CD8^+^ subset expressing (A) CD107a, (B) IFN-γ or (C) TNF-α after restimulation with αCD3 for 18 h. (D–F) Proportion of each CD4^+^ subset expressing (D) CD107a, (E) IFN-γ or (F) TNF-α after restimulation with αCD3 for 18 h. Significance was determined using a 1-way ANOVA adjusted for multiple comparisons, *n* = 11. *P* < 0.05*, *P* < 0.001**, *P* < 0.0001***.

As expected, only a small proportion (<5%) of CD8^+^ T_N_ and T_CM_ expressed CD107a, IFN-γ or TNF-α upon restimulation, indicating they had limited effector function (Fig. 6A–C). In contrast, T_EM_, T_RM_ and T_E_ produced larger amounts of CD107a, IFN-γ, and TNF-α upon restimulation (Fig. 6A–C). T_RM_ expressed the largest amount of CD107a (36%), however, this was not statistically significantly different than the amount of CD107a produced by T_E_ (31%) or T_EM_ (21%, Fig. 6A). Likewise, TNF-α production was higher in T_RM_ (21%), than in T_E_ (15%) and T_EM_ (13%, Fig. 6B), but the differences were not significant. While IFN-γ production was not different between T_RM_ (28%) and T_E_ (22%), T_RM_ produced significantly more IFN-γ than T_EM_ (14%) upon restimulation (*P* = 0.008, Fig. 6C). These results demonstrate that T_RM_ within MPE have greater, or at least equivalent functionality as T_E_ and T_EM_.

A similar analysis was conducted examining the small number of CD4 T_RM_. CD4^+^ T_N_ or T_CM_ expressed no CD107a or cytokines upon restimulation (Fig. 6D–F). A small frequency of T_E_ (22%), T_EM_ (9%) and T_RM_ (16%) expressed CD107a upon restimulation (Fig. 6D) with no significant difference in the proportion of T_RM_ producing CD107a compared to T_EM_ (*P* = 0.06) and TE (*P* = 0.8). A significantly higher proportion of T_RM_ (27% and 33%, respectively) expressed both IFN-y (*P* = 0.003) and TNF-α (*P* = 0.013) than T_EM_ (13% and 22% respectively, Fig. 6E and F). There was no difference in the effector function of CD4^+^ T_RM_ and T_E_ (18% and 21%) with equivalent frequencies of each subset producing IFN-y (*P* = 0.37) and TNF-α (*P* = 0.2).

### CD69 and not CD103 expression on CD8^+^ TRM is associated with IFN-γ production in MPE

In our previous flow cytometry analysis of CD8^+^ T_RM_ isolated from lung cancer tumors, there was complete co-expression of CD69 and CD103.^14^ In contrast, as described above (Fig. 1I), only about 25% of CD8^+^ MPE T_RM_ expressed both CD69 and CD103, with 25% of the cells expressing only CD103 and the remaining majority expressing only CD69. We were thus interested in determining if there were any phenotypic or functional implications of CD69 and/or CD103 expression.

**Figure 7.**
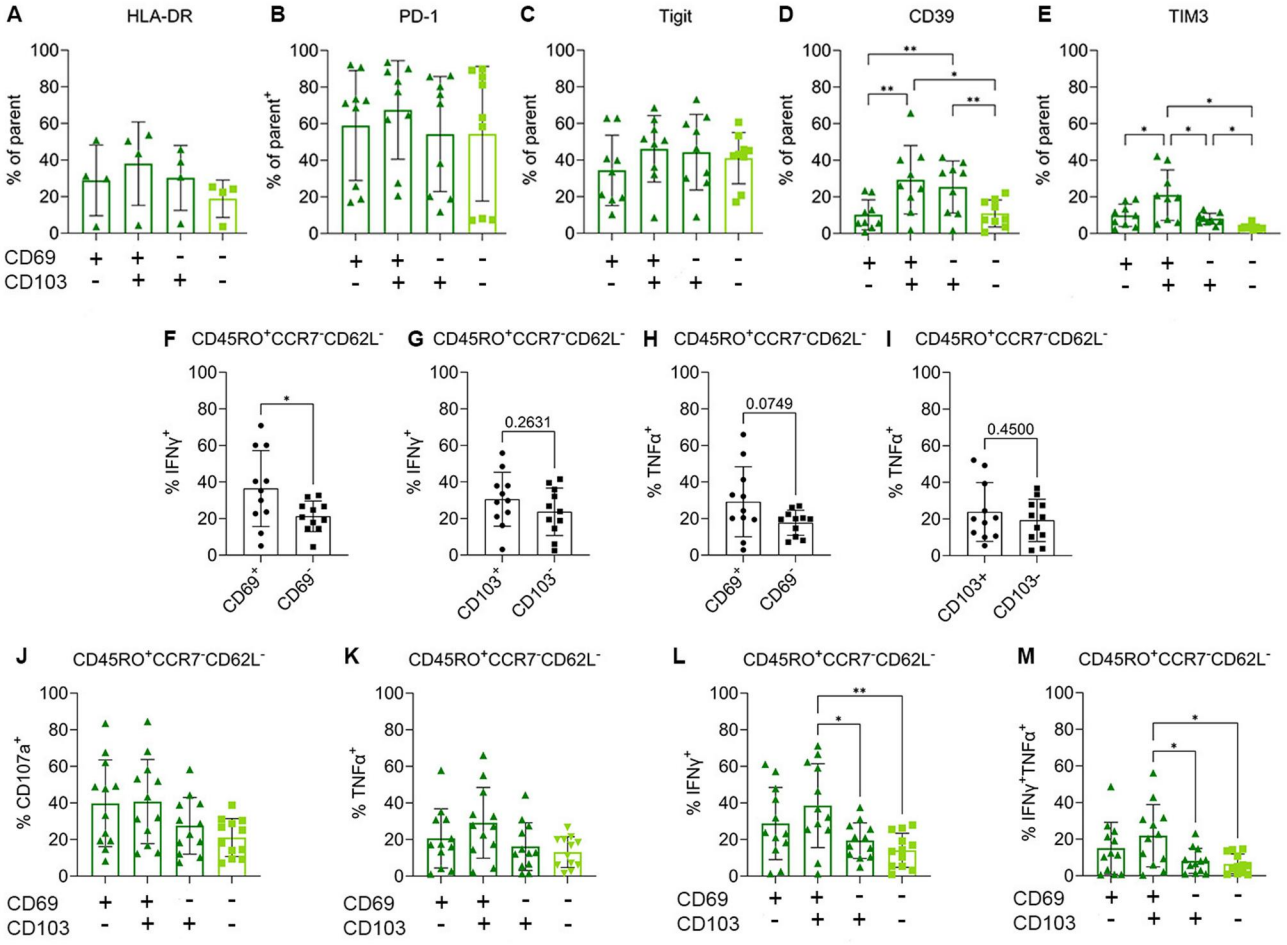
CD69 expression on CD8^+^ T_RM_ is associated with IFN-γ production. (A–E) Expression of activation and inhibitory markers on CD45RO^+^CCR7^-^CD62L^-^ CD8^+^ T cells separated by CD69 and CD103 positivity. (F-G) Percentage IFN-γ^+^ of CD45RO^+^CCR7^-^CD62L^-^ CD8^+^ separated by CD69 (F) or CD103 (G) expression. (H and I) Percentage TNF-α^+^ of CD45RO^+^CCR7^-^CD62L^-^ CD8^+^ separated by CD69 (H) or CD103 (I) expression. (J) Expression of CD107a on CD45RO^+^CCR7^-^CD62L^-^ CD8^+^ T cells separated by CD69 and CD103 positivity (K–M) Expression of IFN-γ, TNF0α or both IFN-γ and TNF-α on CD45RO^+^CCR7^−^CD62L^−^ CD8^+^ T cells separated by CD69 and CD103 positivity. *n* = 4 for HLA-DR, *n* = 9 for all other markers, *n* = 11 for CD107, IFN-γ and TNF-α expression. Significance was determined using a 1-way ANOVA adjusted for multiple comparisons. *P* < 0.05*, *P* < 0.001**.

We first assessed the expression of activation and inhibitory markers on these 4 subsets of T_RM_. There were no significant differences in the expression of HLA-DR, PD-1, or TIGIT depending on CD69 or CD103 expression (Fig. 7A–C). Interestingly, CD39 expression was significantly higher on the T_RM_ expressing CD103 (CD103^+^CD69^+^ and CD103^+^CD69^-^ T_RM_ vs their CD103^-^ counterparts, Fig. 7D). TIM3 expression also varied, with a significantly higher frequency of CD103^+^CD69^+^ cells expressing TIM3 compared to the single positive or double negative T cells (Fig. 7E).

In other studies, CD69 and CD103 expression on T_RM_ has been associated with effector function.^15^^,^^30^^,^^31^ Therefore, we were interested in whether CD69 or CD103 expression on T_RM_ from MPE was associated with the effector function of these T cells.

We first looked broadly at whether CD69^+^ or CD103^+^ memory CD8 cells (CD45RO^+^CCR7^−^CD62L) were more likely to produce IFN-γ and TNF-α after restimulation. A significantly higher proportion of CD69^+^ cells expressed IFNγ then CD69^-^ cells, 34% compared to 21% respectively (*P* = 0.036, Fig. 7F). IFN-γ production was similar between CD103^+^ and CD103^−^ cells (*P* = 0.26, Fig. 7G). There was also a trend towards a higher frequency of CD69^+^ cells (29%) producing TNFα compared to CD69^−^ cells (18%) though this difference was not quite significant (*P* = 0.075, Fig. 7H). As with IFNg, there was no association between TNF-α production and CD103 expression (*P* = 0.45, Fig. 7I).

When co-expression of CD103 and CD69 was examined with regard to effector function, there was a trend towards CD103^-^CD69^+^ and CD103^+^CD69^+^ T_RM_ having a higher effector function then their CD69^-^ counterparts (Fig. 7J–M). CD107a and TNF-α production trended higher in T_RM_ that expressed CD69 compared to CD69^-^ T_RM_ and T_EM_ (CD103^-^CD69^-^) though this difference was not significant (Fig. 7J–K).

There were differences in the frequency of IFNg producing T cells after restimulation based on CD103 and CD69 positivity. A higher proportion of CD103^+^CD69^+^ double positive T_RM_ expressed IFNg compared to CD103^+^CD69^−^ (*P* = 0.015) and CD103^-^CD69^−^ T cells (*P* = 0.001, Fig. 7L). More CD103^+^CD69^+^ T_RM_ also produced both IFNg and TNF-α compared to the CD103^+^CD69^-^ and CD103^-^CD69^-^ subpopulations (*P* = 0.034 and *P* = 0.014 respectively, Fig. 7M).

**Figure 8.**
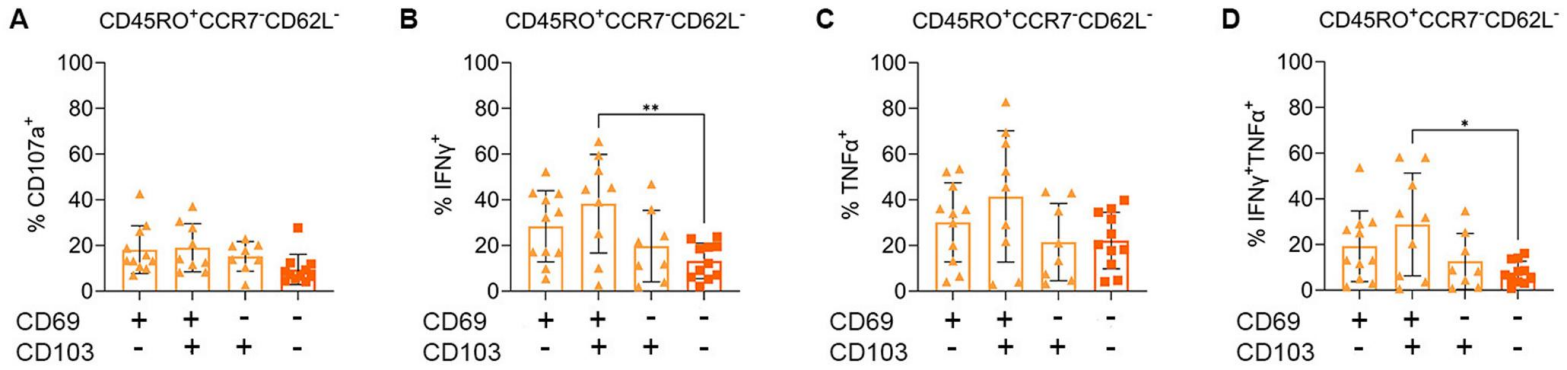
CD4^+^ CD103 CD69 double positive T_RM_ from MPE produce cytokines after restimulation at a higher proportion then single positive or double negative populations. (A–D) Proportion of each CD4^+^ T_RM_ subset expressing CD107a (A), IFN-γ (B) or TNF-α (C) or both IFN-γ and TNF-α (D) after restimulation with αCD3 divided by CD69 and CD103 expression. Significance was determined using a one-way ANOVA adjusted for multiple comparisons, *n* = 11, *P* < 0.05*, *P* < 0.001**.

The effector function of CD4^+^ T_RM_ was also examined (Fig. 8). There was no association between CD107a and CD69 and CD103 expression (Fig. 8A). A higher proportion of CD69^+^CD103^+^ cells expressed IFN-γ compared the double negative T_EM_ CD4 T cell subset (Fig. 8B). While there was no difference in TNF-α production between the populations (Fig. 8C), a higher proportion of CD69^+^CD103^+^ T_RM_ produced both cytokines compared to the double negative population (Fig. 8D).

These data demonstrate that CD69 expression on T_RM_ is associated with increased effector function, with a higher proportion of CD69^+^ T_RM_ producing IFNg after restimulation when compared to their CD69^−^ counterparts; CD103 positivity did not appear to be associated with increased cytokine production.

## Discussion

Consistent with previous studies, we observed that lung cancer MPE contained large numbers of CD3^+^ T cells, skewed largely towards CD4^+^ T cells.^22^^,^^29^^,^^32^^,^^33^ We found that the nearly 80% of CD4^+^ T cells and half of the CD8^+^ T cells expressed CD45RO, indicating a memory phenotype, in line with the literature that reports a higher proportion of memory T cells in MPE that is seen blood.^29^^,^^33^^,^^34^ However, this percentage of memory cells is lower than what is observed in lung cancer tumor-infiltrating lymphocytes (TILs), where almost all of the T cells have a memory phenotype.^14^ Thus, the number of naïve cells in MPEs is much higher than in TILs. This observation is important when considering T cell activation studies in which unfractionated T cells have been analyzed because the naïve T cells in MPE will make the T cells appear “hypofunctional.”

T_RM_ appear at different frequencies in different anatomical locations. Although initially identified in mucosal organs (such as the skin, GI tract, and lung), they have subsequently been found in many other normal locations, presumably poised to rapidly react with infectious agents. Interestingly, T_RM_ are often seen in large numbers in tumors where it has been proposed that a subset of the T_RM_ react with tumor antigens. For example, although the numbers vary somewhat, in lung cancer, it has been reported that between 30%–60% of CD8 TILs and 5%–40% of CD4 TILs express CD69 and/or CD103 and were categorized as T_RM_.^14^^,^^15^ The main goal of this study was to determine if T_RM_ were also present in malignant pleural effusions, and if so, to characterize their phenotype and function using both flow cytometry and single cell RNA sequencing.

Using the surface markers CD103 and CD69, we first employed flow cytometry to identify a population of T_RM_. In our sample of lung cancer effusions, within the memory T cell population, approximately 15% of the CD8^+^ cells expressed CD69 and/or CD103. Approximately 5% of CD4^+^ memory T cells had these T_RM_ markers. There are limited studies that have previously identified T_RM_ in MPE. In comparison to our study, Mao et al. describe expression of the T_RM_ associated markers CD103 and CD69 on 55% of CD8^+^ T cells and 15% of CD4^+^ T cells from malignant pleural effusions and peritoneal ascites.^29^ A previous study that examined T_RM_ in ascites fluid from ovarian cancer patients reported that between 20% and 70% of CD8^+^ cells were CD103^+^, but with a large amount of heterogeneity.^35^

With regard to the phenotype of both CD8^+^ and CD4^+^ MPE T_RM_ using flow cytometry and single cell mRNA sequencing, the expression of the classically described T_RM_ signature genes were, in general, similar to that previously reported in other locations.^25^^,^^28^ Specifically, in addition to lack of CD62L and CCR7 (which mediate lymph node homing) and the presence of CD103 and CD69 (which would favor tissue retention), we noted increased levels of mRNA for ITGA1 (CD49a), TIGIT, LAG3, GZMB, PRF1 and very low levels of SIPR1, KLF2, LEF1, TCF7, and IL7R. By flow cytometry, the T_RM_ expressed relatively high levels of PD1, TIGIT, TIM3, and CD39, similar to what was seen on other memory T cell subsets (T_CM_, T_EM_). While the CD8^+^ T_RM_ lacked expression of some canonical T_RM_ genes, such as RUNX3, CXCR6 and CD69, we could detect expression of these genes within the CD4^+^ T_RM_ population and at levels high enough to differentiate these cells from other T cell subtypes. It has been demonstrated that the effector function of T cells is diminished in MPE compared to the peripheral blood, with these cells often displaying a more exhausted phenotype characterized by decreased secretion of cytokines after restimulation and increased expression of immune checkpoint markers.^33^^,^^36^ However, the functionality of T cell subsets within MPE had not been compared. We found that both CD4^+^ and CD8^+^ T_RM_ had equivalent effector function (as measured by cytokine release after restimulation), as did T_E_ and T_EM_.

CD103^+^ tumor infiltrating lymphocytes, often with a T_RM_ like phenotype, have been associated with an improved prognosis in several cancer types.^15^^,^^35^ In non-small lung cancer patients, CD4^+^ T cells that expressed CD103 had the highest cytotoxic capability, expressing more IFN-γ and TNF-α then other T cell subsets, while within the CD8^+^ subsets, there was no difference in effector function associated with CD103 or CD69 expression.^30^ However, another study reported that CD103 expressing CD8^+^ TILs had increased granzyme B and CD107a expression compared CD103^-^ T_RM_.^15^ Within MPE, we found that expression of CD69, but not CD103, within the T_RM_ populations (CD45RO^+^CCR7^-^ CD62L^-^) was associated with a higher proportion of T cells expressing IFN-γ and TNF-α after restimulation with anti-CD3 in vitro. This finding is consistent with a study that examined T_RM_ in tuberculous pleural effusions^31^, where CD69^+^CD8^+^ T cells, but not CD103^+^CD8^+^, produced high levels of IFN-*γ* after treatment with tuberculous antigen.

While these T_RM_ were obtained from MPE associated with primary lung cancer, they showed marked phenotypic differences to T_RM_ present within lung tumors; primarily, differences in expression of CD103. Studies have reported that 50% to 80% of CD8^+^ T_RM_ in lung tumors expressed CD103, while in MPE we only observed ∼20% of CD45RO^+^CD62L^−^CCR7^−^ CD8^+^ T cells expressing CD103.^8^^,^^37^^,^^38^ Likewise, our previous study found that 60% of CD45RO^+^ CD8^+^ lung cancer TILs expressed CD103^14^. CD69 and CD103 are also highly co-expressed on T_RM_ from lung cancers, however we only saw co-expression on 10% of CD4^+^ T_RM_ and 20% of CD8^+^ T_RM_. TRM within both locations, primary tumor and MPE, did retain effector function despite high expression of inhibitory markers.^14^^,^^37^

This study has several limitations and raises many questions. It must be noted that it is not certain that the expression of CD103 and CD69 on CD45RO^-^CD62L^-^CCR7^-^ T cells is sufficient to define these cells as T_RM_ using flow cytometry,^39^ as demonstrated with the discordance in the frequency of T_RM_ identified in our samples using flow cytometry and single cell RNA sequencing. Furthermore, a universal T_RM_ signature may not exist, as the expression of genes that are canonically associated with migration and tissue residency can be driven by factors not relating to their differentiation into a tissue resident cell.^39^ The number of cases is relatively small and would benefit from larger studies examining lung cancer and other types of MPE so that correlations between cellular composition of the MPEs could be studied. Although our findings show that T_RM_ are a major subpopulation of T cells within lung cancer MPE that have strong effector function, their clinical importance has not yet been established. For example, we do not yet know if the numbers of T_RM_ correlate with prognosis or if the presence of T_RM_ might predict responses to immuno- or other types of therapy. Another major unknown is the specificity of the T_RM_ in MPE. Without determining the clonotypes of the T cells, it remains unclear if most of these cells are specific for various infectious agents (bacterial or viral) or if they are targeting tumor neoantigens. There are some data to suggest that CD39^+^ T cells are tumor-specific.^40^ In our previous study of lung cancer TILs, we found that an average of ∼30% of CD8 T cells were CD39^+^, with a much smaller percentage of T cells in adjacent normal lung being positive.^13^ In the MPE in our study, approximately 20% of the T_RM_ expressed CD39 and may thus be potentially tumor reactive.

In summary, we examined T_RM_ in MPE from lung cancer patients using both scRNAseq and flow cytometry. We found that ∼14% of CD8^+^ T cells and ∼5% of CD4^+^ T cells had a T_RM_ phenotype and expressed one or both of CD69 and CD103 along with genes classically associated with T_RM_ such as ITGA1 (CD49a), GZMB, PRF1 and very low levels of genes for SIPR1 and KLF2. When the functionality of these MPE T_RM_ was examined, we found they had equivalent effector function when compared to T-effector cells (T_E_) and T-effector memory cells (T_EM_), as measured by production of CD107a, IFN-γ and TNF-α after restimulation. T_RM_ expressed comparable levels of exhaustion and activation markers as T_EM_ and T_E_. Finally, we looked at the association between effector function and expression of CD103 and CD69 and found an association of increased function with expression of CD69, but not CD103. Given their importance in controlling tumor growth and responses in other tumors, T_RM_ likely play a similar role in MPE and should be studied further.

## Supplementary Material

vlaf013_Supplementary_Data

## Data Availability

The data underlying this article will be shared on reasonable request to the corresponding author.
