# Supplementary material for: Identification and characterization of tissue resident memory T cells in malignant pleural effusions associated with non-small cell lung cancer
Source: Immunohorizons. 2025 Apr 24;9(6):vlaf013. doi: 10.1093/immhor/vlaf013 (PMC12032394; doi:10.1093/immhor/vlaf013)
Supplement: vlaf013_Supplementary_Data [file vlaf013_supplementary_data.pdf]

Supplementary Figs

Supplementary Table I. Treatment that each patient received prior to collection of MPE.

Pt #	Therapy at the time of thoracentesis
1	Cox inhibitor and pembrolizumab
2	Untreated
3	Carboplatin, pemetrexed, pembrolizumab
4	Untreated
5	Osimertinib
6	Abraxane
7	Osimertinib
8	Untreated
9	Untreated
10	Osimertinib
11	Osimertinib
12	Carboplatin, pemetrexed, pembrolizumab

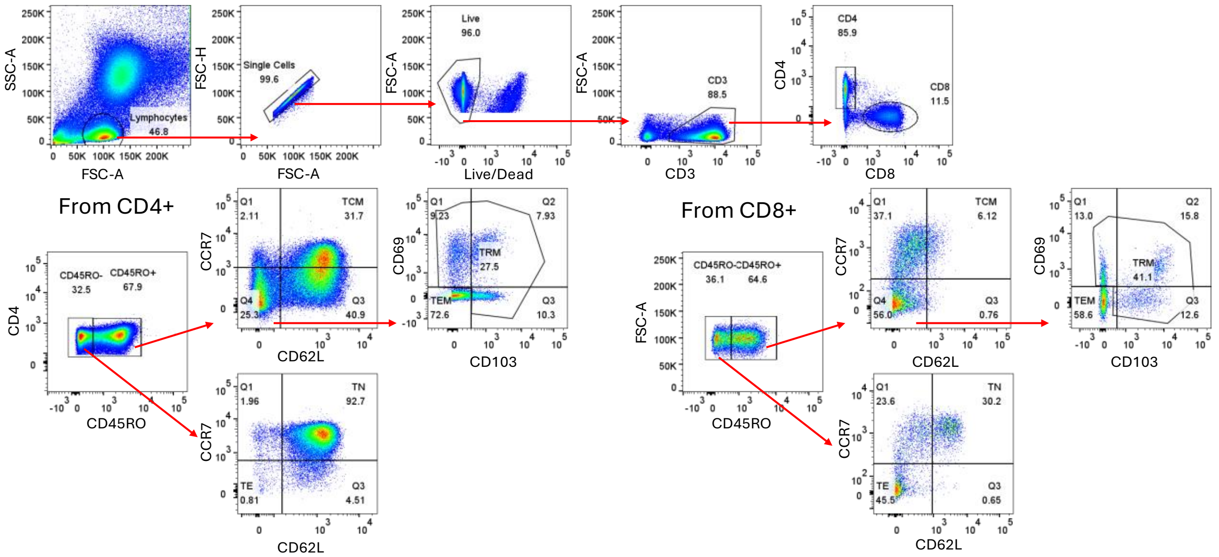

Supplementary Fig 1. Gating strategy for MPE and T cell subsets. Samples were first gated on lymphocytes followed by singlets, live cells, CD3^{+} T cells and then CD4^{+} or CD8^{+} T cells. CD4^{+} and CD8 $^{+}$ T cells were separated into CD45RO^{-} and CD45RO^{+}. T_N were classified as CD45RO^{-}CCR7^{+}CD62L^{+}, T_E as CD45RO^{-}CCR7^{-}CD62L^{-} and T_{CM} as CD45RO^{+}CCR7^{+}CCR7^{+}. Within the CD45RO^{+}CCR7^{-}CD62L^{-} population, cells were gated on CD103 and CD69. T_{EM} were identified as CD45RO^{+}CCR7^{-}CD62L^{-}CD103^{-}CD69^{-} and T_{RM} broadly as CD45RO^{+}CCR7^{-}CD62L^{-} and expressing one or both of CD103 or CD69.

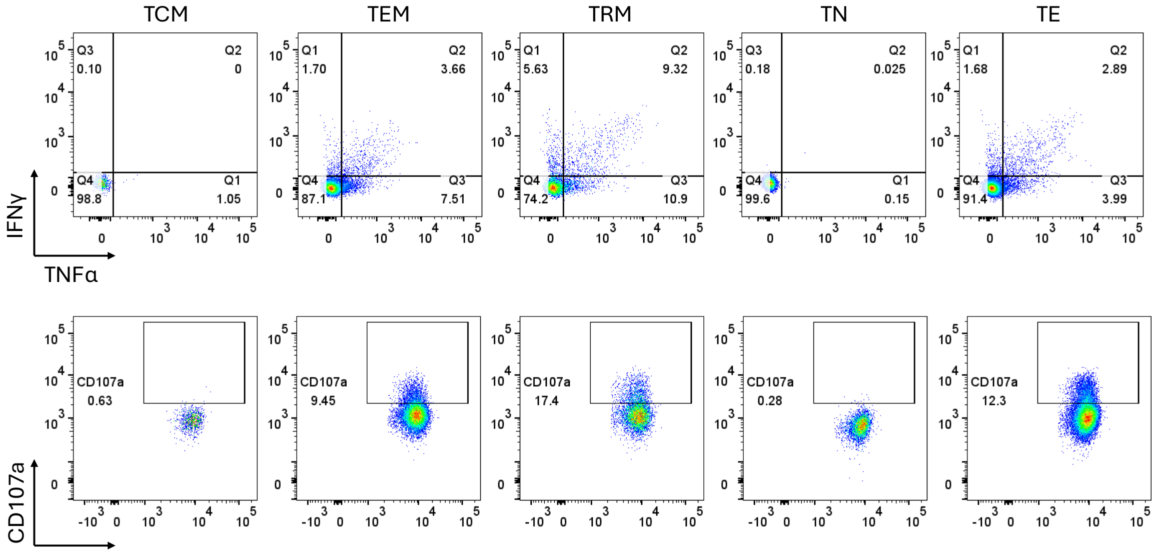

Supplementary Fig 2. Representative flow cytometry plots for CD8$^+$ T cell populations showing IFNγ, TNFα and CD107a expression after restimulation. Samples were first gated on lymphocytes followed by singlets, live cells, CD3$^+$ T cells and then CD8$^+$ T cells. Cells were then gated into T cell subsets as in supplementary figure 1. Gates were set an using unstimulated control.

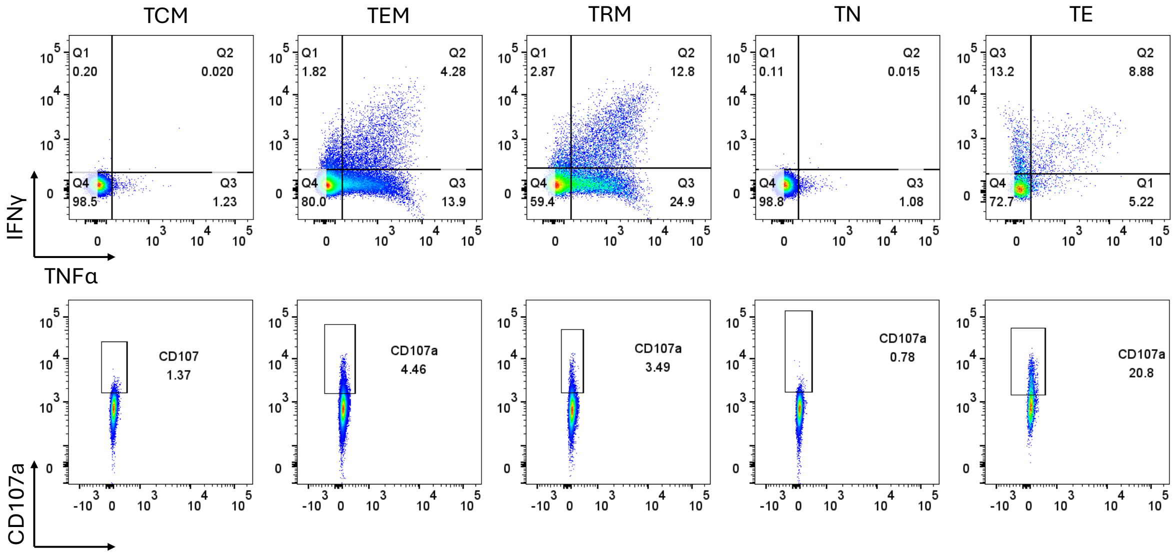

Supplementary Fig 3. Representative flow cytometry plots for CD4$^+$ T cell populations showing IFNγ, TNFα and CD107a expression after restimulation. Samples were first gated on lymphocytes followed by singlets, live cells, CD3$^+$ T cells and then CD4$^+$ T cells. Cells were then gated into T cell subsets as in supplementary figure 1. Gates were set an using unstimulated control.
